# Supplementary material for: Employed mothers’ breastfeeding: Exploring breastfeeding experience of employed mothers in different work environments in Ethiopia
Source: PLoS One. 2021 Nov 12;16(11):e0259831. doi: 10.1371/journal.pone.0259831 (PMC8589202; doi:10.1371/journal.pone.0259831)
Supplement: S1 File — (DOCX) [file pone.0259831.s001.docx]

**Participant Information and Interview Guide**

**Participant information sheet**

Title– Employed mothers’ breastfeeding: Exploring breastfeeding experience of employed mothers in different work environments in Ethiopia

Hello, my name is Firmaye Bogale, I am working on a study on employed mothers’ breastfeeding experience of employed mothers in different work environments in Ethiopia.

The aim of this study is to explore and compare the breastfeeding practices of employed mothers with 6 months maternity leave, employed mothers who have access to onsite child care center and mothers with neither of the accommodations and to explore the experience.

You might find this research interesting in that it tries to cover an issue that is crucial and unavoidable to any mother specially who is working and anyone who has a relation to a working mother and also shows the rarely seen side of this issue, and in that the result will help mothers, children and the society as a whole.

The disadvantage might be that it could cause some discomfort as it asks you to deeply explain about your unique individual experiences. However, I have tried to eliminate this by making it anonymous and confidential. If you agree to take part in this study we will have a discussion on some questions I have regarding your breastfeeding experience as honest and free as possible for this research to fulfill its objective. And it should be known that your participation in this project is entirely voluntary and you are not obliged to participate in any way and if you don’t want to participate you don’t have to give a reason and you can withdraw any time you wish to.

**Consent form**

I have been informed about the purpose and use of this research and the information I am going to give will be used only for the purpose of this study and my identity as well as the information I provide will be kept confidential.

And so

1. I agree to participate in this research voluntarily ------------------

2. I don’t agree to participate in this research ------------------------

Researcher- Firmaye Bogale

Email- fbfbogale93@gmail.com

Phone no- 0913754332 75

**Interview guide**

**Mothers in depth interview**

Date of interview ____________

Time started ____________

Facility ______________

Time ended ___________

**Introduction**

I am working on a research which is mainly concerned with child breastfeeding and working environment for breastfeeding mothers in Addis Ababa city. I thought you are one of the most resource full people in this regard because you are an employed mother who has a young breastfeeding child. I would appreciate it if you would talk to me.

**Mother**

Age of the mother __________________

Marriage status ______________________

Working position/ the name or title of your job/ __________________

Years of schooling/college _____________

**Children**

Total number of children _____________________

Number of under-five children _________________

Gender of the youngest child __________________________

Age of the child ____________________________

**Section one: Knowledge and attitude pertaining to breastfeeding**

1. What led you to make the decision to breastfeed?
2. For how long do you think a child should be breastfed?
3. What kinds of benefits do you or your child receive because of breastfeeding?
4. For how long do you intend to breastfeed your baby?

**Section two: Returning to work**

1. Before you returned to work, I’m sure you had some thoughts or plans as to what it would be like to breastfeed or pump when you went back to work.
2. How did you plan to continue to provide breast milk to your baby when you returned to work?
3. Let us talk about the reality of what it was like for you to breastfeed or pump when you returned to work after having your baby?

Probe- what were the challenges?

**Section three: Experience with breastfeeding at work**

1. What were the first few days like for you when you returned to work?

Probe- child care, leaking

1. How did returning to work impact your breastfeeding decisions?
2. Regarding to breastfeeding what was the biggest concern you had when you returned to work?
3. Can you tell me what challenges you faced while trying to breastfeed after returning to work?
4. What did you do to prepare for returning to work and to continue to breastfeed?
5. Who at work did you speak to about the possibility of breastfeeding/pumping at work?
6. How did breastfeeding continuation after returning to work affect your job?
7. What kind of support did you have to breastfeed or pump when you returned to work?

Probe- What kind of support did you have from family, partner, and friends?

1. What do your co-workers and employer think of you breastfeeding after returning to work?
2. Can you tell objections you faced because of continuing breastfeeding after returning to work? And also from whom those objections came from?
3. Can you explain to me what kinds of challenges you faced during breastfeeding after returning to work?
4. What kinds of facilities are fulfilled at your work place that enables you to continue breastfeeding? Such as a pump, refrigerator, breastfeeding breaks, and comfortable lactation room?
5. What is it like to use those things at work?
6. As a working mother who wants to continue to provide breast milk to her baby can you tell me what support would be helpful to you?

**Section four: Knowledge of right and mother friendly program guidelines**

1. What would you think of a program that encourages businesses to become breastfeeding friendly and support mothers?

Probe- Giving longer maternity leave, having flexible work schedules to provide time for expression of milk and providing access to hygienic storage alternatives for the mother to store her breast milk?

1. How does this compare to your experience?

Probe- what is different? What is similar?

1. How would having your company publicize this information impact your breastfeeding decisions?
2. How realistic is this for the business you currently work for?

Probe- Why do you say that?

1. How do you think working for a company that supported breastfeeding in that way would impact your breastfeeding choices?

**Section five: Interview on Maternity leave**

1. When did you inform your employer that you were pregnant and Can you explain what happened when you told him/her?

Probe- what was his/her reaction

1. How long was your maternity leave?
2. How did you find out about your maternity leave?
3. When did your maternity leave start? Before/ after you gave birth?
4. What do you think of breastfeeding and maternity leave relation?
5. For how long did you give your child only breast milk?

**Section six: For women with six months maternity leave**

1. What opportunities has the six months maternity leave created for you with respect to breastfeeding?
2. What challenges have you faced because of the six months maternity leave?

Probe- career wise, from your colleagues

1. Can you explain how you continue to breastfeed after returning to work?
2. What difference would it bring if you have access to your child every day during work hours?

**Section seven: For women who have access to onsite childcare center**

1. How did you find out that there is onsite childcare center here and from whom? 2-3-
2. Do you breastfeed at the center?
3. Are you comfortable breastfeeding at the center?

Probe- If no, what is the reason

1. What facilities are made available to assist you?

Probe- washing facilities, private room, nanny/nurse, comfortable sitting arrangement

1. Do you have breastfeeding breaks?
2. If yes

Probe- a) Are those breaks considered as working time/ paid?

b) Length of time every day

1. What challenges do you face while breastfeeding in the center?
2. Can you explain to me how the presence of onsite childcare center affects your breastfeeding behavior?
3. What additional changes and interventions do you think would improve your breastfeeding experience?
4. What additional changes and interventions do you think would improve your breastfeeding experience?
