# Supplementary material for: Employed mothers’ breastfeeding: Exploring breastfeeding experience of employed mothers in different work environments in Ethiopia
Source: PLoS One. 2021 Nov 12;16(11):e0259831. doi: 10.1371/journal.pone.0259831 (PMC8589202; doi:10.1371/journal.pone.0259831)
Supplement: S2 File — (DOCX) [file pone.0259831.s002.docx]

**Themes, Sub-themes and Codes**

Employed mothers’ breastfeeding: Exploring breastfeeding experience of employed mothers in different work environments in Ethiopia

| **Theme** | **Subtheme** | **Code** | **Quotes example** |
| --- | --- | --- | --- |
| **Workplace barriers and facilitators to breastfeeding** | Past experience | *Past effect* | *The fact that I didn't breastfeed my older baby pushed me to breastfeed my younger one. My older baby used to get sick all the time and I used to take her to the clinic repeatedly. But thanks to God my younger baby is healthy and I don't take her to the clinic often. Mn* |
|  |  | *Past effect* | *oh, the difference is that the younger one can resist any sickness but the older one gets really sick even with cold and because of this I used to take her to different places to get her checked and she has poor appetite. Mn* |
|  |  | *Past effect* | *Surely it has advantage in every aspect. Not only for her health but also for strength. My younger baby is stronger than my older one and the older one is really skinny. Mn* |
|  |  | *Past effect* | *As I’ve told you before it (breastfeeding) is good for my child health and growth and I’m happy because I feel like I’ve fulfilled my responsibility as a mother. Mn* |
|  |  | *Past effect* | *For children to grow and prevents disease and for me, it makes me happy and it is made for them to breastfeed so I think it is important to breastfeed. Msm* |
|  |  | *Past effect* | *It is good for my child’s health, for his brain and everything and for me it increases my love for him and I feel happy when I breastfeed. Mcc* |
|  | work challenges to the point of quitting | *Emotional stress* | *I can’t put my whole focus on work; I get frustrated because my child doesn’t drink well and I think he might get hungry or cry while I am here. I feel sad and sometimes even want to quit my job because I’ve no choices.* Mn |
|  |  | *Emotional stress* | *Before I brought my child to the center, I was stressed about how she would stay the whole day without breastfeeding and I was worried that she might fall or get hurt. Everything about her made me stressed but it all stopped once I brought her to the center and everything got better. Mcc* |
|  |  | *Emotional stress* | *You can’t put your whole focus here, you get frustrated plus your breast gets really full and some babies wont bottle feed. So it is hard. Mn* |
|  |  | *Emotional stress* | *You leave your baby for a maid at home but you call the maid a 100 times to remind her to change the diaper and to give him the milk you left and specially because the maids usually don’t know much about children I call her to bottle feed him every two hours and to change his diaper and to freshen him up. Mn* |
|  |  | *Emotional stress* | *I used to think that she might get hungry and get stressed but it all stopped once I brought her here. The thought of my baby getting hurt. I was stressed about how she would stay the whole day without breastfeeding and I was worried that she might feel down and everything about her made me stressed. Mcc* |
|  |  | *Physical challenge* | *After I spend my day at work I get tired and breastfeeding after that seems like another tiring job to do which even makes me mad. The other thing is after I spend the whole day at work my breast becomes full and becomes painful to even breastfeed while the breast is engorged. Mn* |
|  |  | *Physical challenge* | *I got sick the first day I worked a night shift my breast was hurting me so I went home right after I finished my job and I stayed home feeding her the whole day. I was even going to stop breastfeeding her at all since I started working night shift. Mn* |
|  |  | *Physical challenge* | *because I don’t breastfeed in the day time I try to compensate that by giving he more in the night time and I can’t wait to eat something in the morning so I know it hurts me. Mn* |
|  |  | *Physical challenge* | *Sometimes you might be late for work because he wants to breastfeed and would not let go especially as they get older they get more eager to breastfeed. Mn* |
|  |  | *Physical challenge* | *It was difficult. Your breast gets full and painful. I couldn’t return in two months because I developed infection after operation so I stopped after that Mn.* |
|  |  | *Emotional challenge* | *Forget the other things I didn’t even feel like a mother with my first child because I didn’t breast feed her I raised her with other foods and other things. I used to get jealous of other people breastfeeding their babies and giving them love looking in to their eyes.Mn* |
|  |  | *Physical challenge* | *For instance I used to go out after half day of work and that was taken from my annual leave but there are also other workers like health workers that go out like me but I don’t know if their annual leave was also taken, actually mine would not have been taken if it wasn’t for the situation of my colleagues. Mn* |
|  |  | *Physical challenge* | *It was very difficult. My breast used to get full and spill so I had to bring breast pump with me and it was really hard, motherhood is not easy. I couldn’t be attentive at my work and so it was hard to work with full orientation so it has impact on work too because when my breast gets full and painful all I could think of was going home and breastfeeding. Mn* |
|  |  | *Physical challenge* | *It was ok. I didn’t have that much of difficulty because I stayed around three weeks after finishing the six months I started giving my child other foods and my breast got full sometimes but it was ok because I go home in the afternoon. So I didn’t have that much difficulty. Msm* |
|  |  | *Effect on work* | *Yes I mean customers will not be served well when you go out to breastfeed and they will have to stay for you to come back. And one time a customer was not served well and had a problem with work. Mn* |
|  |  | *Effect on work* | *Of course the first thing is a lot of stress because of not breastfeeding your child this is because you leave a child who doesn’t take anything other than breast milk which makes you think all the time so you can’t give a 100% to your job so there might be a gap on your work and because I don’t find transportation easily when I come back from lunch I come in late sometimes so clients might stay for a while here so it has impact in these ways. Mn* |
|  |  | *Employer help* | *I, fortunately, had a good boss and he used to let me go home and breastfeed with some intervals and I knew he would allow me to do that. If that was not the case I would quit my job. Mn* |
|  |  | *Employer help* | *We have breastfeeding breaks to breastfeed and check on our children. We come here at 10 am and 3 pm to breastfeed our children and we get back to work after 30 minutes. The presence of such a situation has made the problems easier. Mcc* |
|  |  | *Employer help* | *There is no payment cut. This is done to increase women’s participation. We come here at 4 and 9 o’clock to breastfeed our children and get back to work after. When we come here we let our bosses know and there is no problem. Mcc* |
|  |  | *Employed help* | *I fortunately had a good boss and he used to let me go home and breastfeed with some intervals and I knew he would let me do that. But if this was not the case I would have stopped working. Mn* |
|  |  | *Employer help* | *They support me to continue breastfeeding. I’m not talking about this organization. I’m talking about the earlier bureau I worked in. my boss and everyone else used to support me, there were other mothers there so we used to discuss about breastfeeding and how we should breastfeed for the sake of our children’s health. Mn* |
|  |  | *Employer help* | *After I got back to work I used to go out for half-day to breastfeed which was, of course, unofficial and done with only agreeing with my boss as most mothers do. But the difference is that mine lasted shorter. And later I was told that it will be deducted from my annual leave which is not the case for some other mother but I don't understand why such differential treatment prevails. Mn* |
|  |  | *Leave effect* | *It was hard. As you know maternity leave in our country is 3 months. I took my annual leave and used it for half day every day for some time but then I breastfed him only at night and when I get off work. But the 3 months is not enough it is very short. Mn* |
|  |  | *Leave effect* | *because I think the government allows only two months maternity leave and when the mother stays at work the whole day the milk content of the breast will also decrease it won’t be as before there are mothers who wean early because their breast stops having milk. Mn* |
|  |  | *Leave effect* | *What changed is the fact that my child couldn’t get breast milk because of me getting back to work. He doesn’t breastfeed when he wants to as before and the amount of my breast milk is also decreasing from time to time because he is not breastfeeding as much as before. Mn* |
|  | Supporting condition at work | *Organizational support* | *It was good because the caring we have in this organization is immense. The first thing is what this organization has made for us has made us love one another and our kids more plus we come here to see our children in our working hour and that expense is covered by the organization does not have any impact on our payment no cuts or nothing. We come in here in the morning and the afternoon to breastfeed and see our children but there is not even a penny taken from us because of it. Mcc* |
|  |  | *Organizational support* | *I spill my breast milk in the toilet when it gets full. It will be contaminated even if I express it here because there is nothing to store my milk in.* Mn |
|  |  | *Organizational support* | *There is no problem with the leave here. I even took additional leave after I finished my maternity leave and I stayed home for around 7 months. We are even allowed to come in late at 2 in the afternoon, 30 minutes more for breastfeeding. Msm* |
|  |  | *Organizational support* | *I have no words to describe it. I’m telling you ther truth it makes me feel happy because I am a mother and I love the children very much---(crying with happiness)--- I used to work here even before I gave birth and I used to be so close with the children here so the other mothers saw that I love kids so much so they gave me a present as a thank you. Mcc* |
|  |  | *Organizational support* | *It is not only me that praises concern with the things done for women because this organization is very supportive of women in different ways including the six months leave and I think it good for other organizations to take this experience and use it for themselves. Msm* |
|  |  | *Organizational support* | *I stayed home for three months and breastfed my baby well. But after I resumed my work I started giving her formula milk which I don’t think is healthy. But I had to since I didn’t have additional support.* Mn |
|  |  | *Organizational support* | *I previously left my children home at three or four months with a maid and I used to worry about them a lot. I had to give my babies formula milk early to get them used to it because I worry they might get hurt if I don’t. But it is different now. Msm* |
|  |  | *Organizational support* | *In addition to the six months leave they supported me by giving me the annual leave I had and sometimes I might come late to work because of breastfeeding or other things related with my child but they understand my situation. Msm* |
|  |  | *Organizational support* | *The care provided in this organization is immense. What this organization offered has enabled us to provide more care for our kids since we have the opportunity to see our children in our working hours. This shows how much we are valued by our employer. Mcc* |
|  |  | *Organizational support* | *I'm happy about this center but the one concern I have is that the nanny here is busy because she takes care of all the children and becomes tired. I think she needs help and we have asked for additional baby sitters. Mcc* |
|  |  | *Organizational support* | *I feel lucky that I get to spend the whole day with my baby. I care for all of the children as my own. I think this arrangement works for the children and us the mothers pretty well. Mcc* |
|  |  | *Effect of working environment* | *yes there is a difference I breastfed my first child for about three months and some days and I started giving her food because I had to start work but with my second child I breastfed exclusively for six months and my child didn’t get sick because I stayed home with him he was ok the whole time but my first child used to get sick because I left her home with a maid. After staying with them for six months they will not get that hurt even if you see them only in the morning and evening so staying for six months has a lot of advantage. Msm* |
|  |  | *Effect of working environment* | *Yes. I don’t think I would have breastfed for this long because it is a must to give her other foods if I start working like with my other children where I had to work around fourth month which made me start other food early. Msm* |
|  |  | *Effect of working environment* | *When I gave birth to my first child I was given a 45 days maternity leave and I was working in governmental organization at that time and I took my annual leave and also added a sick leave and extended my leave to three months and because my home and working place was close to each other I started complementary feeding at four months but now I exclusively breastfed for the whole six months. Msm* |
|  | View on working environment | *Maternity leave application* | *We are advised to exclusively breastfeed up to 6 months for our children to remain healthy. We cannot do that because we return to the office just after 3months.* *Mn* |
|  |  | *Facility to breastfeed* | *I spill my breast milk when it gets full on the ground because I think it would be contaminated and there is nothing here to store the milk like a fridge. I know there are people who send their milk home for someone with a car for them to drop it at home. Mn* |
|  |  | *Facility to breastfeed* | *I would have fed him if there was a fridge to store the milk. Mn* |
|  |  | *Support* | *Because as I have told you my breast milk was decreasing in amount and there is nothing comfortable here to express like a fridge where we can keep the milk or a room where we can express in so I didn’t think of expressing because of these reasons. Msm* |
|  |  | *Maternity leave application* | *The thing is that there needs to be the willingness of the organization to take all of my leave after birth. For example, if I am told to take one month before giving birth as a rule, then I can't do anything about it. Some organizations are flexible and allow mothers to take their three months' maternity leave after giving birth which would motivate us. Mn* |
|  |  | *Maternity leave application* | *The first thing is everywhere we go we are told to breastfeed for six months but the leave we are given is three months, no wait, what they put is for us to use two months post birth which is not fair and something that doesn’t go together. Mn* |
|  |  | *Maternity leave application* | *No no, I even got the three months because I was in agreement with my boss but when you see the situation here whether you use the 1 month prenatal leave or not you are left with only two months of post natal maternity leave. So three months is not enough when you think of breastfeeding let alone two months. Mn* |
|  |  | *Support* | *If there is an onsite CCC you will not be stressed and you can breastfeed your baby frequently because the child will be with you. Both your job and child will be benefited and we (mothers) will be healthy and refreshed. I say it is an excellent option. Mn* |
|  |  | *Support* | *My child used to be shy but now she is not afraid of people and she wants to play with everyone, she has become confident. There is nothing that could hurt them here it is comfortable and not dangerous at all. Mcc* |
|  |  | *Support* | *In my opinion, CCC brings a lot of hustle to mothers. You have to bring your baby in the morning and return in the evening which is a lot of work. We come from far so it is exhausting both for the mother and child. I also think it will be hard to give 100 percent with your baby being close by. So I'm more inclined to having six months' maternity leave. Mn* |
|  |  | *Support* | *I gave my child formula milk at 3 months because I had only three months' leave and that is bad for my baby. So having six months leave would mean complying with the recommendation of six months of exclusive breastfeeding. Mn* |
|  |  | *Support* | *To say that I could continue to breastfeed without this leave is a joke. If I didn’t have this leave I would have started giving my baby other foods early and she would not have been this happy or attentive. Mn* |
|  |  | *Support* | *What I think is that currently we see and hear in different medias that a mother should breastfeed exclusively until six months but how is an employed women supposed to do that? So I support the implementation of six months leave proclamation. Mn* |
|  |  | *Support* | *Well, organizations might not benefit much from this (six months' maternity leave) so what will happen is that women's employment will decrease in time. Giving six months would be challenging for some businesses and consequently, the organizations may lose the interest to hire women. Mn* |
| **Coping mechanisms** | Annual leave as a key | *Alternative* | *I knew I would not be able to work effectively or breastfeed my child properly if I came in to work at three months. So I had to use my entire annual leave to stay longer. You know people don't have to be a woman to feel our pain, everybody should understand that the three months is not enough by any measure. Mn* |
|  |  | *Alternative* | *Well after I used my maternity leave I used the annual leave I had because there is nothing one wouldn’t do for their child and because I was given sick leave for about six weeks all those added up and became six months so I breastfed my baby for six months and got back to work. Mn* |
|  |  | *Alternative* | *I think the breastfeeding break is better because you can go home and breastfeed your baby freely and the bond between mother and child will increase too and also our breast keeps on producing milk more if the baby is breastfeeding but if we keep on expressing it all the time the milk will decrease and expressing is not as easy as people think it is painful. So I think it is a good idea to give mothers breastfeeding breaks both for the mother’s comfort and her child’s health. Mn* |
|  |  | *Alternative* | *I knew If I came in to work at three months I would not have been able to work effectively or breastfeed my child properly so I had to use all my annual leave to stay longer. Mn* |
|  |  | *Alternative* | *For example as I have told you earlier it would be good if there was a place to express milk and a fridge to store the milk and a breastfeeding corner too. So I think it would be good if we have these things in the future. Mn* |
|  |  | *Alternative* | *We have it really good here and I think it is better to have a six months leave better than a three months leave with a daycare center because I see many friends of mine who are given three months leave but I notice that they stay for five months or so by adding their annual leave or using a sick leave so one way or another they will stay longer but it would be effective if they are given the leave officially. Msm* |
|  | lasting resolution | *Recommendation* | *The change should come nationally from the government or else organizations and bureaus won't allow it and improvement in the EBF rate would not be practical. Mn* |
|  |  | *Recommendation* | *We have the privilege of getting child care center because we work in an international organization but our government has to work on this and do this for all and the three months is not enough for instance I have a neighbor who had to take one month prenatal leave and get back to work two months after giving birth before baptizing her daughter which is really sad so I think the maternity leave should be six months and for the future. Mcc* |
|  |  | *Recommendation* | *I think it would be really good if maternity leave becomes six months for all women because three months is a short time other organizations should learn from this organization. Msm* |
