# Supplementary material for: Employed mothers’ breastfeeding: Exploring breastfeeding experience of employed mothers in different work environments in Ethiopia
Source: PLoS One. 2021 Nov 12;16(11):e0259831. doi: 10.1371/journal.pone.0259831 (PMC8589202; doi:10.1371/journal.pone.0259831)
Supplement: S3 File — (DOCX) [file pone.0259831.s003.docx]

**Interview data**

**Employed mothers’ breastfeeding: Exploring breastfeeding experience of employed mothers in different work environments in Ethiopia**

**1/Motivation**

**Past experience others experience**

MN

01-oh, the difference is that the younger one can resist any sickness but the older one gets really sick even with cold and because of this I used to take her to different places to get her checked and she has poor appetite.

But my younger one has a good appetite and a good body weight that I don’t even look at her with my full eyes. But my older child doesn’t eat much she even started eating injera after she turned 4 I think she finds it to be sour. But now she is starting to by looking at her younger sister.

The fact that I didn’t breastfeed my older baby really pushed me to breastfeed my younger one. I used to take my older baby to the clinic many times each month and she used to vomit often and she used to get sick all the time. But thanks to God my younger baby is really healthy.

Forget the other things I didn’t even feel like a mother with my first child because I didn’t breast feed her I raised her with other foods and other things. I used to get jealous of other people breastfeeding their babies and giving them love looking in to their eyes.

surely it has advantage in every aspect. Not only for her health but also for strength. My younger baby is stronger than my older one and the older one is really skinny.

A- well I saw it with my older child she became more vulnerable to disease and she couldn’t even handle a cold because she didn’t breastfeed. I was told to breastfeed her when she gets sick and that there is no other treatment for her and I gave her hot milk for her when I get home. But for my younger child I only give her breast milk and I don’t take her to the clinic often and when I breastfeed her every thing becomes fine.

07-The things I see on my child are way better than those who don’t breastfeed. He doesn’t get sick from time to time and he is strong so I breastfeed him because it makes me happy and he breastfeeds well too.

1. As I’ve told you it is good for his health and many other things and I’m happy because **I feel like I’ve fulfilled my responsibility.**
2. I think breast milk is meant to be given for the baby so why keep it in? and I don’t want my baby to be just like other children who don’t breastfeed for instance I have a niece who stopped breastfeeding at the age of two months and she gets sick repeatedly and I think he’ll get sick too.

MO

O2-It is good for my child’s health, for his brain and everything and for me it increases my love for him and I feel happy when I breastfeed.

MS

02- For children to grow and prevents disease and for me, it makes me happy and it is made for them to breastfeed so I think it is important to breastfeed.

04-Yes! She breastfed because I have been told that colostrums is important from hospital and I knew from the past because of IYCF project in concern and I also had awareness when I gave birth to my first child.

**Knowledge and mothers perception**

MN

02- For my child I don’t think she would get better if she got sic. Generally I don’t even think she will grow I feel like her growth would be different than my first child. If she doesn’t breast feed

03- I think what helped me most is the fact that I’m working in a health facility and because I hear a lot of good things about breastfeeding and I plan to breastfeed for two years

Breastfeeding feels good to the mother and children love it and I also think it makes them strong. Though I’m not sure I think I’ve heard something about protection from cancer but I breastfeed more because I think it helps my child.

breastfeed her because I know it helps her with her growth, health and her mental development. I believe I have to do anything to help my child even if I get hurt.

06- It is very useful for my child to prevent abdominal cramp for example if I give him other additional things in the middle. The first thing is ahe would be vulnerable to disease and second is that she can’t resist disease and third is the fact that essential for health and growth and it might have use for me like preventing cancer.

If my baby doesn’t breastfeed I might get sick of mastitis because the milk that should have been out stays in and turns in to puss and blood causing infection and I believe she should breastfeed or else she’ll have a higher chance of being malnourished

07- I feel like I’ve fulfilled my responsibility.

I think breast milk is meant to be given for the baby so why keep it in

08- Because I am told to breastfeed during visits to health center and its use is told through different media Radio, TV and many more. So looking at its advantage I understood that I had to breastfeed

Well my baby is strong and it has helped him gain weight plus my love for my baby has increased very much because of breastfeeding. And it has helped me to lose the weight I gained during pregnancy.

MO

03- Usually people breastfeed for a year and a half but I think it should be for two years.

02-Two years. Longer if possible because it is good for his body according to science. And people say that it is good for a women to breastfeed

MS

01- I breastfed her because it’s said repeatedly by radio and different medias that it is good for children to be breastfed with in the first one hour and that is what I have been told during my checkups too but because I had an operation I couldn’t give her within an hour but I hear that it is good for the growth of a child.

I have heard that it is very important for children and that it well balanced with different nutrients so that is what helped me to continue breastfeeding.

02- I have been told to breastfeed during my visits and I hear about it on different medias so I am breastfeeding.

03- And I think it is better for me to breastfeed rather than the milk staying in my breast.

It would not be good for her growth and I think it is better for my breast milk to be fed than stay in. I feel like it would have a problem if it stays in.

04- The importance. I know that it important for my child’s health, growth and for her future life so that is what made me breastfeed.

It is clear what the use is for my child. It makes her healthy, she would have a balanced diet, the chance of getting sick will decrease and she will have a good growth so it is essential.And for me the major thing is the mental satisfaction I get and in addition to that it helps me to physically be healthy and fit and decreases the chance of having a breast cancer.

**Influence**

MN

01- My first baby didn’t breastfeed at all though I tried hard to feed her again and again so she grew up by feeding other milk with a bottle not breast milk and I was also going to do the same with my last baby when my friends came to see me they told me that linseed helps with making breast milk spill faster and they were right and my baby started breastfeeding.

I knew this from before. My mom used to tell me that she fed us (her children) up to 4-5 years and that I should not stop early so I think it should be for at least two years with other foods

I gave her water with “tenaadam” because it is said to be good for babies. And I also gave her formula milk “nun” but she didn’t take that well and my friends also told me that breast feeding is a must so I started giving her then

02- Well, when we go to health facilities we are advised to breastfeed at least eight times a day so I think a child should get accordingly. And during my maternity leave I tried to breastfeed my baby as much as I could and I’m still trying to.

MO

01- It is currently highly emphasized I hear about it everywhere plus my mom tells me to breastfeed my child all the time even after I gave birth and I hear from different medias that it is important for children.

**Mothers belief (-ve)**

MN

01- milk we give them after working a night shift is going to make them sick.

04- I mean I can breastfeed her up to six months but it depends on the kind of food i eat because mostly breast milk decreases in time maybe If the mother takes good food the child may not get hurt too but I prefer if the child is given something to complement them like a cow’s milk or formula milk around three or four months.

05- There are things I hear plus breastfeeding has many advantages to my baby so I choose to breastfeed.

Excuse me but I don’t believe in the six month you publicize. i mean the breast might not have enough milk even if we eat well. I think three months is enough I also started formula milk at three months when I got to work.

It prevents me from different diseases for instance it prevents cancer. And my child will get things he wouldn’t normally get like the things I eat and drink and it’s also good for his health and growth.

I knew from the start that I had to breastfeed for my baby to grow healthy. I didn’t need convincing I held it as a slogan so I didn’t discuss it with any one and I didn’t listen to any one telling me other wise.

MO

03- I don’t know exactly but I think three months is enough. It is better if additional food is started after that. I gave my baby breast milk only for three months then she started formula milk

**Societal influence -ve**

MN

01-But sometimes it is believed that children tend to not eat food when they keep on breastfeeding; it makes them lose appetite for other food types. And we don’t think that breast milk has much use, we are more inclined to giving food and cow milk leaving our breast behind for them to wean and this is also because it is not feasible with our job(tan color)

**Past experience**

MN

Caution none found

**Uncertainties**

MN

01-Up to six months without anything but what would happen if your breast is not coming out what would happen to the baby? You can’t do anything you will just feed him other food

MS

1. I think if the mother’s breast keeps on producing milk it would be good if a child breastfeeds longer but sometimes it dries out early and that makes it hard( uncertainities

**Challenge by baby**

MN

1. 08-sometimes you might be late for work because he wants to breastfeed and would not let go specially as they get older they get more eager to breastfeed.

**Challenges on women related with work and breastfeeding**

**Emotional stress**

MN

03- You cant put your whole focus here, you get frustrated plus your breast gets really full and some babies wont bottle feed. So it is hard.

Well it’s my baby. I get stressed because he doesn’t bottle feed well and things like him being hungry, crying and irritable makes me stressed.

Well I feel sad but I’ve no choice other than to accept the reality. There is no choice until the law for six months leave gets implemented

04- I used to miss my baby I sometimes even want to leave my job and be with my baby.

I was worries that she would die off hunger and I was afraid that she would get hurt. that was my biggest concern.

05- You leave your baby for a maid at home but you call the maid a 100 times to remind her to change the diaper and to give him the milk you left and specially because the maids usually don’t know much about children I call her to bottle feed him every two hours and to change his diaper and to freshen him up.

Specially his health, when I breastfeed my baby at home I breastfeed him after washing and cleaning my breast but when you use feeding bottle it can get dirt on it……he could get sick if they give him that and the other thing is, I was stressed about how he was going to get used to the maid that it was all ----

07- I used to get stressed and feel guilty because I take what he could feed from him. I used to express and leave it at home but he finishes that fast so I get very stressed.

08- so I carry all this burden and my life is filled with rush I think of my child when I am at work and run to work when I am at home there is not even a comfortable situation to breastfeed my child for a short minute and because of all this I can’t breastfeed freely.

MO

03- I used to think that she might get hungry and get stressed but it all stopped once I brought her here.

the thought of my baby getting hurt. I was stressed about how she would stay the whole day without breastfeeding and I was worried that she might feel down and everything about her made me stressed.

MS

01- Because I know that breastfeeding should be continued as a complementary after six months I was stress how it would be because I get busy at work and I know it would not be possible to go out that often so I thought that I would breastfeed her at night.

It was very hard. I used to miss my baby and I wanted to just go home and be with her and I didn’t work with full attention at first it was very hard at first.

I was stressed because I knew I couldn’t go out as much as I like to see my baby because our job makes us busy but It would have been even harder if she hadn’t started breastfeeding less and .

03- I didn’t think anyone could take care of my child as I could so that bothered me.

**Physical challenge**

MN

1. I got sick the first day I worked a night shift my breast was hurting me so I went home right after I finished my job and I stayed home feeding her the whole day. I was even going to stop breastfeeding her at all since I started working night shift

I feel pain when I feed her, because my breast gets full and tauten when I stay at work I find it to be really painful to breastfeed her and I sometimes even want to stop her from breastfeeding in the middle. It is after she breastfeeds a little bit that the pain goes away

When you spend the day at work you will be tired and breastfeeding after that seems like another tiring thing to do so you just want to rest and it even makes you mad to breastfeed after that

02- because I don’t breastfeed in the day time I try to compensate that by giving he more in the night time and I can’t wait to eat something in the morning so I know it hurts me

because she used to breastfeed well and get full. We both used to get enough sleep but now it’s the reverse. It is like she tries to compensate and she doesn’t want to let go od the breast even when she is sleeping. And you’ll be amazed how she grabs on to the breast even when I try to take it out of her mouth.

03-when you get home your breast gets tauten and is painful If he is breastfed. When it is full but this would not happen if he breastfeeds in time

My breast used to get full and painful plus my cloth used to get wet and ruined it was beyond words

But on the second day my breast got really full and because of that it became painful so the option I had was to spill the milk and so I used that method for some days

03-You can’t put your whole focus here, you get frustrated plus your breast gets really full and some babies wont bottle feed.

04- I pill it on the ground because I think it would be contaminated and there is nothing here to store the milk like a fridge . I know there are people who send their milk home for some one with a car for them to drop it at home.

06- because employed women like me go to work at 12 in the morning and back at 12 in the evening that means you stay 12 hours without looking after your baby.

And also my breast gets full, painful and my close gets ruined.

07-It was difficult. Your breast gets full and painful.

I couldn’t return in two months because I developed infection after operation so I stopped after that.

08- It was very difficult. My breast used to get full and spill so I had to bring breast pump with me and it was really hard, motherhood is not easy. I couldn’t be attentive at my work and so it was hard to work with full orientation so it has impact on work too because when my breast gets full and painful all I could think of was going home and breastfeeding. I couldn’t focus on my job it was a big problem.

What concerned me the most was my breast I’m not going lie. I stressed about what I was going to do because even before coming back to work my breast used to get full and spill around 4 o’clock after I breastfeed him in the morning so I was concerned how I could handle it after returning to work

For instance the first problem is transportation and second is that my breast gets really full and ruins my close so I feel ashamed when I go home

04- It was ok. I didn’t have that much of difficulty because I stayed around three weeks after finishing the six months I started giving my child other foods and my breast got full sometimes but it was ok because I go home in the afternoon. So I didn’t have that much difficulty.

1. It is hard. your breast becomes full and painful, it spills and ruins your close and it is not a good feeling but you’ll get used to it through time and I got better faster because my breast milk was starting to decrease because I came back after six months and three weeks.

**Work related challenges**

MN

04- Yes I mean customers will not be served well when you go out to breastfeed and they will have to stay for you to come back. And one time a customer was not served well and had a problem with work.

08- But eventually my breast milk started decreasing I think that it is because my child doesn’t breastfeed repeatedly because I stay at work and I also thought of my baby getting hurt because he couldn’t breastfeed as much as he needs too. But I try to go home and breastfeed in my lunch break despite the challenges from my work place. But generally there is a great problem.

Third is that if I leave work early even with five minutes every one notices you and different complains come your way saying that clients are not served well and generally there is no understanding

Of course the first thing is a lot of stress because of not breastfeeding your child this is because you leave a child who doesn’t take anything other than breast milk which makes you think all the time so you can’t give a 100% to your job so there might be a gap on your work and because I don’t find transportation easily when I come back from lunch I come in late sometimes so clients might stay for a while here so it has impact in these ways.

**3/Effect of working environment**

**Comparison**

MN

04- It of course has effect preciously I leave my children hoem at three or four months with a maid and I used to worry about them a lot I used to think is they had eaten or I might find them crying or sick but now I can see my baby every day here with me and all the mothers care for all the kids as their own.

And with regard to breastfeeding, because I had to leave my children and get back to work I give my babies formula milk early to get them used to it because I think they might get hurt if I don’t-

02-yes there is a difference I breastfed my first child for about three months and some days and I started giving her food because I had to start work but with my second child I breastfed exclusively for six months and my child didn’t get sick because I stayed home with him he was ok the whole time but my first child used to get sick because I left her home with a maid. After staying with them for six months they will not get that hurt even if you see them only in the morning and evening so staying for six months has a lot of advantage.(comparison)

03- Yes. I don’t think I would have breastfed for this long because it is a must to give her other foods if I start working like with my other children where I had to work around fourth month which made me start other food early.

04- When I gave birth to my first child I was given a 45 days maternity leave and I was working in governmental organization at that time and I took my annual leave and also added a sick leave and extended my leave to three months and because my home and working place was close to each other I started complementary feeding at four months but now I exclusively breastfed for the whole six months.

**Leave effect**

MN

01- Well it is advised to exclusively breastfeed at least for six months and when we get back to work after three months maternity leave our children will be forced to stop breastfeeding.

if you at least breastfeed your child for six months you would feel that he/she has breastfed enough.

I stayed home for the three months and because I was with her every time she wanted to breastfeed very much then I started giving her formula milk when I got back to work

03- It was hard. As you know maternity leave in our country is 3 months. I took my annual leave and used it for half day every day for some time but then I breastfed him only at night and when I get off work. But the 3 months is not enough it is very short.

I breastfed him exclusively aWhen I was home until my maternity leave was over. That is for a little more than 3 months then it was a must to start formula milk.

04 Well he doesn’t get the milk in a timely manner, every 2 hours. If I was at home I would give him every 2 hours but when I am at work I stay here for 8 hours so he doesn’t breastfeed that much he gets hurt. He gets what he is supposed to with formula milk.

06- If the leave is short and your home is far for instance, you’ll give your baby extra things other than breast milk it is a must

07- because I think the government allows only two months maternity leave and when the mother stays at work the whole day the milk content of the breast will also decrease it won’t be as before there are mothers who wean early because their breast stops having milk.

08- What changed is the fact that my child couldn’t get breast milk because of me getting back to work. He doesn’t breastfeed when he wants to as before and the amount of my breast milk is also decreasing from time to time because he is not breastfeeding as much as before.

example I had to use all my annual leave to stay longer but at three months only I know how much I used to suffer from back pain so If I came in to work at that time I would not have been able to work effectively or breastfeed my child properly. You know people don’t really have to be a woman to feel our pain everybody should understand and the three months is not enough by any measure

**Fit to work**

MN

1. *The thing that has changed since I started staying at work is that my breast doesn’t hold as much milk as it used to. Part of it is because I don’t eat or drink as much when I am at work*
2. *Now, I don’t have a constant interval of breastfeeding. I breastfeed as much as I can. I breastfeed whenever I can because I can’t schedule because of work*.

MO

1. *while at home I used to breastfeed her whenever she wanted repeatedly but after I came here it changed to only after I got back to work and in the morning before I coming here and my breast milk had started decreasing in amount and* it might have dried if I hadn’t brought her here and continued breastfeeding

**Organizational support**

MO

02-It was good because the caring we have in this organization is immense. The first thing is what this organization has made for us has made us love one another and our kids more plus we come here to see our children in our working hour and that expense is covered by the organization does not have any impact on our payment no cuts or nothing. We come in here in the morning and the afternoon to breastfeed and see our children but there is not even a penny taken from us because of it.

- **I have no words to describe it. I’m telling you ther truth it makes me feel happy because I am a mother and I love the children very much---(crying with happiness)--- I used to work here even before I gave birth and I used to be so close with the children here so the other mothers saw that I love kids so much so they gave me a present as a thank you and**

**Generally the value that this organization gives for mothers and women is beyond words for me. So I want to thank the owner of this organization by the name of the mothers the children for what he has done for us (unique**

**Sometimes people ask me where I keep my baby and tell me they would pay any price because of the difficulty they face but when I tell them about this place they get shocked and tell me how luck I am so i see how much people suffer and it makes me thank God and honor this organization even more and I don’t think I would have a problem even if I had ten children with this organization and this center in place**. **(Unique help)**

MS

02-It is not only me that praises concern with the things done for women because this organization is very supportive of women in different ways including the six months leave and I think it good for other organizations to take this experience and use it for themselves.

04- very. Not only for employees but breastfeeding is also promoted in any area that concern works at.

**view of working situation**

**CONTRADICTING**

MN

Well it is advised to exclusively breastfeed at least for six months and when we get back to work after three months maternity leave our children will be forced to stop breastfeeding

01- I mean we are told to at least breastfeed exclusively until six months by health professionals and for that to be true we need to be together with our children. But we can’t do that if we are working the child has to be started on other milk or foods.

02-. I even expected that it would happen before I gave birth but it’s not implemented yet. It is said that a child should be breastfed up to 6 months or the child will get hurt. On the other hand they are telling us to get back to work only after 3months which is contrary to each other

05- What I think is that currently we see and hear in different medias that a mother should breasstfeed exclusively until six months but how is an employed women supposed to do that? So I support the implementation of six months leave proclamation.

with which most organizations are governed with that states prenatal leave of 1 months duration. I would be happy if this is corrected because with this proclamation a mother gets back to work when her baby is two months old and she’ll use her annual leave if she has any but if she doesn’t have that she stays home the whole one month home before giving birth so she breastfeeds for only 2 months despite the six months recommended.

06-No, this should come nationally or else organizations and bureaus won’t allow it and this would not be practical.???

08- It has storng relation the first thing is everywhere we go we are told to breastfeed for six months but the leave we are given is three months, no wait what they put is for us to use two months post birth which is not fair and something that doesn’t go together

It not even three months when you see it for real it is two months after birth so it should be taken as a two months maternity leave.

These days we hear a lot about women health and how it is mandatory to empower women but besides the propaganda we haven’t seen anything in action so we want these things to happen in reality

**Implementation**

MN

04- I have question on this prenatal and postnatal leave, in this organization and in all the others there is a protocol that states that a mother should take one month leave before birth, of course it is true that she will be at risk after the 8^th^ month but for women who can work till birth their gets wasted without use. For instance I have a friend who lost her one month leave because she was told to get back to her job after 2 months of maternity leave. I think it would have been good if this is not the situation. This is what I think.

05- No no, I even got the three months because I was in agreement with my boss but when you see the situation here whether you use the 1 month prenatal leave or not you are left with only two months of post natal maternity leave. So three months is not enough when you think of breastfeeding let alone two months.

Between me and the organization, there needs to be the willingness of the organization because it is clearly stated on the proclamation and if you are told to go accordingly. then it’s your responsibility but if they agree then it is fine and it would motivate female employees.

06- I had three months. One month before birth and two months after but fortunately I was able to stay at work until I gave birth so I only took one day before birth, I used the whole three months after birth.

07- I was given two months leave after birth. I had one month leave I didn’t use before pregnancy but it was not possible to post pone it to after birth.

08- I took the three months by my will because it was a short time I wanted to take it after there was no obligation.

MO

03- I was getting tired because I work on a machine and I was also told that I had one month before birth and two months after birth so I had to use it. So it was both willingly and mandatory.

**Onsite +ve**

MN

1. I support the idea of onsite child care center. I mean I don’t know if my husband’s mother is going to stay for long or not and that’s why his mother always tells me to have a baby again while she still has the strength to care for a baby. And if his mother wasn’t here I would not be able to come back to work I would have to wait till my baby gets in to school so I would be happy about the child care center idea.

And the six months maternity leave is not enough so I prefer the onsite child care center even if it is around the bureau and not in the compound of the bureau.

05- The other thing is if there was a child care center just like some organizations we would be able to breastfeed and care for our children.

08 A- this is also a good idea the fact that you can watch after your child by itself give you satisfaction and it is better than a place just for expressing milk because if you keep on expressing for longer time the breast milk decreases in amount so it is better to promote the onsite childcare center more.

A- I think it is a good idea. When there is a child care center your child will come to work with you so you will not be stressed and you can breastfeed your baby frequently with two hour interval and both your job and child will be benefited and mother will also be healthy and refreshed so I say it is a good idea.

MS

01-I think it would be really good I mean the fact that I could see my child here gives me a mental relief and i could work properly. For instance I leave my baby at home with a maid and I stress about her all the time I call home so many times but if she was here near me I would not have been stressed and I could have worked effectively (support from longer).

MO

03- Let me tell you what happened one day, I stayed the whole day at work and I got home late because of traffic jam but when I got home my child was crying so hard I think she feel down or something hit her and I got so mad and I got in to a fight with the maid it was hard. But after I started bringing her here thanks to god I haven’t seen any problem except for low fever but at home she used to get sick a lot I used to be afraid that I would lose my baby. After I brought her she has a better growth she has started walking now. If I had known I would have brought her earlier and she would have grown even faster than this so I’m really happy about this center and she has also started breastfeeding better than before.

here the only downside is that the kids might feel cold while bringing them in the morning but once she gets here she starts playing and eats better. For instance my child used to be shy but noe she Is not afraid of people ahe wants to play with every one ahe is confident now.

it is peaceful here there is no electricity that they could get hurt with and even if they want to get out of the room it is comfortable and not dangerous as you can see.

oh it was difficult. My breast used to get full and spill and ruin my close it was hard at first then I started getting used to it then after I brought my baby here there is no problem because I breastfeed her twice.

it might have dried if I hadn’t brought her here and continued breastfeeding

01- It has allowed me to check on him and breastfeed well in a timely manner but if he was at home I would not breastfeed him regularly so it has a lot of advantage.

Every body tells me that I am lucky and sometimes people don’t believe me that it is done by the expense of the company they think that we pay and they ask me to show them where I keep my baby.

When I think of what I could have done without this center I find it hard to imagine. To say that I would continue breastfeeding is just unrealistic I mean how could I with my baby at home? I’m very happy that we have this center and it is one of the reasons I gave birth because this is an opportunity I will not get any where so I though that I should use the opportunity. But I don’t know what I could’ve done if we didn’t have this center here.

It feels like home, you put ypur baby here and look after him and breastfeed him. This center has a great role for me in having a baby and continuing to breatfeed.

I feel like I’m at home but if it was other place outside here I’ll be stressed about how he is doing but here all mothers care equally so there is nothing that causes stress or fear.

because if I stay here I get up in the morning knowing I have a job to get to which makes me happy (sense of fulfillment).

I can’t even think of what I would do if I didn’t have access to this center. I would have problem finding a baby sitter to take care of my baby so my husband and my family always thank this organization for creating this center which is not accessible in most organizations. So they are very happy about it

I was a little tired that’s all I had no other problem. I breastfed him when my breast got full because he is here with me. I used to get worked up thinking that he might not sleep at first but once I saw how well they took care of him I got used to it and stopped stressing about him.

It is not enough but it is ok for us because we have an onsite daycare center here. But it would of course be better if we were given a little more time for our children to get a little more matured before coming here.

02- I didn’t think about that too much because this organization has done a big thing for me by building this center and I don’t think other mothers will stress about this too I even think that the presence of this center increases th tendency of giving birth and increases the bond nad love of a mother and a child.

There is no change it is the same. I think I breastfeed him here better because I don’t have to go here and there I just come here in time to breastfeed and give him other additional foods. So there is no change.

Nothing, what do I need if I have my baby with me here? If he was some where else I would have stressed about what he would eat and drink and how he would sleep. But now I see him every day so I didn’t worry about anything.**(less emotional stress)**

I work as a guard of this compound and I love the children here and they love me too and of course I leave my work when I come here to see my baby for 30 minutes but we cover for one another when we come here so there is no big effect on my job. This even made me love my job better and made me work harder. **(Better work)**

The love and respect in this place makes you forget any pain you have and I would be happy if I could talk about this every time. I sometimes want people to ask me where I keep my baby just to talk about this place and the organization. Every body tells me that I am lucky to be in a place like this because there is not a lot of place like this one

But I don’t think it is comparable with the childcare center because here she can breastfeed and take care of him as she wants to and her child will grow with his mother under her protection getting her love. But with the six months leave we should think about what would happen when she gets back to work leaving her baby home so I think the daycare center is better

**Onsite –ve**

MN

01- I think it depends on the mother. You can do both It is similar to working and learning simultaneously you can do your job properly and breastfeed properly too. If you give more focus to your baby than necessary you won’t be able to do your job effectively and you will lose your job.

02- But this is a health facility so I’m afraid that its not good to bring my child here. I’ve never tried to bring my child here I never thought about it.

So I think the government should try to give us six months. That’s it. But the idea of taking children to work place and staying at an onsite child care center is not practical to me.

for instance bringing my child from AddisuGebeya (home) to my bureau is a big problem. I’m afraid of bringing my baby even once a month let alone every day. For example today my baby is feeling ill but I’d rather take her to clinic near my home than bring her here because there might be a lot of suffocation on my way here in a taxi or other transports and she might get sick of a draft. Due to reasons like this I don’t see it as a good option so the better option is six months maternity leave

I’ve said I don’t believe in the onsite childcare center and I would not bring my child. Unless I had no one to look after my baby. But since I have a good baby sitter and since she is drinking the milk well I don’t think I have any reason to bring her to onsite child care center.

06- in my opinion I don’t think the onsite childcare center is not that good specially for health professionals because of their job. For example I work in emergency room and I see patients who are bleeding, unconscious or asthmatic and when I leave to watch my baby some ones life will be at risk here so I think it would be better if the six months maternity leave gets implemented.

It might be good but the longer they stay the more likely that they share different diseases like pneumonia so I don’t think the environment would be comfortable and suitable for children. In my opinion it is better to treat children well at home.

07-- the child care center might have disadvantage because mothers might lose concentration with their baby being close but it will be good if mother fulfills her responsibility of work and take care of her baby too

. Because the child care center has a lot of hustle. You have to bring your baby in the morning and return in the evening . it is tiring both for the mother and child.

I don’t know. In my opinion it is a lot of work because you might come from far and I think the mother won’t give a 100% to her job because everyone gives priority to their child. So I’m more inclined to the six months maternity leave.

**Longer leave**

MN

01- I once heard that the government is going to make maternity leave as long as 6 months. I was very happy to hear that and everybody anticipated what it would mean for mothers.It would be real good if it was six months but even if that doesn’t exactly happenwe need more time for our child that we have been through a lot for. We don’t trust them with anything let alone not breastfeeding added to that.

02- It will have a difference if it is six months. Because I had only three months leave I gave her formula milk at 3 months and that is not advised by doctors. If its possible no other milk or food is needed up to six months of age. So giving us the six months leave would mean putting the advice directly to work.

To strengthen what we’ve talked about, since it’s a science proven thing that children need to breastfeed until six months old to be healthy I think we should stand by it and try to make the maternity leave as long as 6 months. This is all I want to say.

03- I think so. the six months maternity leave is good because you’ll be able to breastfeed your baby and not worry when you come back to work. And you will be well put together for your job so this one is really good.

04-There is no question of that. But mothers are benefited and the thing is that the child will grow up to help his country and a strong and bright child will create something good so I think it is good.

I prefer the six months leave. I would be happy if I treat my baby well for six months and get back to work.

it will affect the baby. Of course I am still breastfeeding my baby but had I been given longer leave I would breastfeed her more. If I stayed home I would breastfeed her the whole day better than now.

05-As I have told you earlier this is not for us the mothers rather for our babies.

Plus if we live close to our work places we would give our child the right foods from the time that he/she should start taking additional foods.

06- Before I gave birth I was hopeful that the maternity leave would be six months but that didn’t happen. It would have been a good solution

I think it is good if the six months gets fixed too because we would be able to take care of our baby and we can arrange what he will start as a complementary and differentiate what he will eat and not eat. And also we can tarin the person that is going to take care of our baby well so I think it’s better because the job gets done and the baby becomes healthy.

I think it would be good if it is six months because science states that a child should get nothing other than breast milk unitl six months and I believe that too. So if it doesn’t hurt the job and studies are done on that six months is preferable.

So if you are given longer time you can raise your child with enough care and you can breastfeed and start complementary food when you get back to work because even starting food and getting your baby used to that is really difficult by itself but if we had time we would be able to make that perfect and get everything in place so it would be better for health too.

What I want to say is that when we talk about maternity leave it should be known that **it’s not for our enjoyment but for our children. Only a mother knows what it feels like to be a mother**

It is safe to leave a baby home at six months because even if the baby doesn’t take expressed milk he/she can wait for you cause she’ll take little bit of everything and the baby starts eating complementary foods

07- But if she can stay home for six months she can start other foods for her child so the six months is good

I think six months is enough for the mother and child because the child will be able to eat other additional foods.

So I’m more inclined to the six months maternity leave.

What I want to say is that I would be really happy if the six months maternity leave gets implemented.

08-What I want to say is that I would be really happy if the six months maternity leave gets implemented.

MS

01- Wow it is very good. It helped me breastfeed as the doctors recommended and the love of my child has increased because of that I used to dedicate all my time to take crae of her and breastfeed her so it is very good. And she has become a little more mature because I came in at six months and she started taking food so it is very important (support longer).

I think the six months leave is better. I think staying the time when you should breastfeed your baby exclusively at home is better because after six months children start to take other foods and breast milk is going to be as a complement. It would be even better if we could have six months leave and then a place here where we can keep our babies. But I think six months is better form the two breastfeeding for six months has more use (support longer).

I have friends who work in other governmental and private organizations and they tell me that it is very difficult for them because they have only three months and they tell me that there are some places where one month prenatal and two months postnatal leave is given so I think we are lucky to have six months

1. It made me breastfeed without the stress of starting complementary food early and I raised my second child with better health than my first.(good longer

There is no negative effect. It is more like I was refreshed and happier when I got back to work and I am working even better not less.

1. The first thing is that it made me breastfeed my child for six months with no worry and it also helped me care for my child until she got a little older than my other children.

The six months definitely. With the six months you get to raise your child better.

04- Yes there is no question. We can breastfeed well with no additional thing and then we can start complementary feeding.

Are you kidding? how could I have continued breastfeeding her if it wasn’t for this leave. If I didn’t have this leave I would have given her other foods early and she would not have been this happy and attentive.

Longer –ve

MN

05-Well as an organization, the organization might be harmed.

What will happen if the leave becomes six months organizations is that organizations won’t hire women or it will decrease. Six months is hard when you see it from the business point so I think the number of women will decrease. Organizations that hire women will decrease.

08. But the disadvantage for the mother is that she won’t be able to share different information that would help her in her career and also advices from her friends on how to feed her child plus she won’t be thinking of herself that much because she will be occupied with her baby. So I think the maternity leave should be longer but should be in the right length of time not too long , not too short

MO

03-- by the way six months is not a lot of time. We breatsfeed up to three months then we might hire a maid and come back to work because a job is a permamnent thing we have to do. But the maids don’t take care of the child as much as we would they don’t fee or take care of our baby

Our job is permamnt but with the six months we can only stay at home with our children for that limited time and then leave them with a maid at home and they might not be well taken care of and there are lots of things that could happen to the children like falling down and getting hurt which my baby can be an example because she got dislocated several times. And there are also children who got burnt because of negligence

**Comment on solution**

MO

03-after I gave birth but I didn’t bring my baby here when I got back I hired a maid at home and made my baby stay at home because my husband didn’t want me to bring her here because he was worried that she would get cold and get sick

- he was not willing at first and he didn’t want me to bring her here because he was worried that she would get sick but as she started getting better here he saw the change and started supporting it.

**Coping mechanism**

**Solution final**

MN

06- No, this should come nationally or else organizations and bureaus won’t allow it and this would not be practical.

**Solution by mom**

MN

01- I work only till six o’clock. I come in the morning and only stay until six o’clock to breastfeed.

I kept on doing that until one month of returning to work after finishing my three months of maternity leave and then I started staying the whole day.

03- I didn’t. I just used the annual leave I had.

04- I used to go home around four in the morning, at lunch and sometimes around 9 in the afternoon then return to work.

After 4 months. Because I used one months of my annual leave.

1. Well after I used my maternity leave I used the annual leave I had because there is nothing one wouldn’t do for their child and because I was given sick leave for about six weeks all those added up and became six months so I breastfed my baby for six months and got back to work.
2. I think the breastfeeding break is better because you can go home and breastfeed your baby freely and the bond between mother and child will increase too and also our breast keeps on producing milk more if the baby is breastfeeding but if we keep on expressing it all the time the milk will decrease and expressing is not as easy as people think it is painful. So I think it is a good idea to give mothers breastfeeding breaks bothe for the mother’s comfort and her child’s health.
3. I knew If I came in to work at three months I would not have been able to work effectively or breastfeed my child properly so I had to use all my annual leave to stay longer

You know people don’t really have to be a woman to feel our pain everybody should understand and the three months is not enough by any measure

MS

01-For example as I have told you earlier it would be good if there was a place to express milk and a fridge to store the milk and a breastfeeding corner too. So I think it would be good if we have these things in the future (additional help).
