# Supplementary material for: Employed mothers’ breastfeeding: Exploring breastfeeding experience of employed mothers in different work environments in Ethiopia
Source: PLoS One. 2021 Nov 12;16(11):e0259831. doi: 10.1371/journal.pone.0259831 (PMC8589202; doi:10.1371/journal.pone.0259831)
Supplement: Attachment — Submitted filename: Review report _D-20-40733.docx [file pone.0259831.s004.docx]

Review of the manuscript: PONE-D-20-40733_reviewer

| Summary | Overall, the manuscript presented an important aspect of child health and wellbeing as well as human rights issue. Through the qualitative descriptive analysis, it has presented factors related to motivation and berries of breastfeeding in working environment nicely. However, there has some issues should be taken care of this manuscript so that readers can understand the information clearly. |
| --- | --- |
| Major issues | No major issues to be addressed |
| Minor issues | a) Line: 75-78 / I think your research questions and specific objective of this manuscript are similar. If not, please mention about the specific objectives.  b) Line: 97-98/ it would be good to make a separate paragraph about interview guide and questions.  c) Line: 101-103 / about the demographic information collection, you could write in the paragraph of participants.  d) Line: 117-122/ This paragraph can be taken as first paragraph for consistency of the information.  e) Line: 128/ did you transcript it in local dialect or translated into other language? Which language ? Please make it clear.  f) Line: 142/ what is Amharic ? if it is local language please make clear the meaning in the bracket.  g) Did you use any qualitative data analysis software for data coding? If you do please mention name and version of the software.  h) Line: 154-155/ 64% mother’s had 1 child; did 64% mother also belong to mean age 30 years? If different please write different line.  What about the status of other percentage participants’ status? You should mention both.  i) Line: 166-169/ Can you give the full abbreviations of the word mn, mcc and msm?  j) Line: 194/ Did women perceived that there previous children were not in good health because of lack of breast feeding. How did they know about it? |
| Other comments | You have collected data from employees of in both government and private organizations . What about the government policy about maternity leave in Addis Ababa? You could mention it in the discussion section. |
