# Supplementary material for: Employed mothers’ breastfeeding: Exploring breastfeeding experience of employed mothers in different work environments in Ethiopia
Source: PLoS One. 2021 Nov 12;16(11):e0259831. doi: 10.1371/journal.pone.0259831 (PMC8589202; doi:10.1371/journal.pone.0259831)
Supplement: Attachment — Submitted filename: Response to Reviewers.docx [file pone.0259831.s006.docx]

**Journal requirements and Authors response:**

1- Please review your reference list to ensure that it is complete and correct. If you have cited papers that have been retracted, please include the rationale for doing so in the manuscript text, or remove these references and replace them with relevant current references. Any changes to the reference list should be mentioned in the rebuttal letter that accompanies your revised manuscript. If you need to cite a retracted article, indicate the article’s retracted status in the References list and also include a citation and full reference for the retraction notice.

**Authors response:** Thank you for the notice and information. We have not included a retracted reference in our manuscript but we have only added references #29 and #30.

**Reviewers' Comments to the Authors and Authors response:**

**Reviewer #2:**

1- The authors have done a great job of responding to the comments. Please copyedit the document.

**Authors response:** Thank you for your time and comments. We have now copyedited the document.

**Reviewer #3:**

1- Thank you for the opportunity to review the updated manuscript titled, "Employed mothers’ breastfeeding: Exploring breastfeeding experience of employed mothers in different work environments in Ethiopia." The authors successfully addressed the majority of reviewer comments. However, the text added to the Introduction to describe the quantitative research done in Ethiopia on the topic is missing citations. Please add citations to support the claims made on lines 79-82.

**Authors response:** Thank you for your comments and your time. We have now inserted relevant references to the statement on lines 79-82
